# An Efficient Direct Position Determination Method for Multiple Strictly Noncircular Sources

**DOI:** 10.3390/s18020324

**Published:** 2018-01-23

**Authors:** Jiexin Yin, Ding Wang, Ying Wu

**Affiliations:** 1 National Digital Switching System Engineering and Technology Research Center, Zhengzhou 450002, China; Cindyin0807@163.com (J.Y.); hnwuying22@163.com (Y.W.); 2 Zhengzhou Information Science and Technology Institute, Zhengzhou 450002, China

**Keywords:** array signal processing, passive localization, direct position determination (DPD), noncircular source, frequency domain, extended subspace data fusion (SDF), Newton-type iteration

## Abstract

This paper focuses on the localization methods for multiple sources received by widely separated arrays. The conventional two-step methods extract measurement parameters and then estimate the positions from them. In the contrast to the conventional two-step methods, direct position determination (DPD) localizes transmitters directly from original sensor outputs without estimating intermediate parameters, resulting in higher location accuracy and avoiding the data association. Existing subspace data fusion (SDF)-based DPD developed in the frequency domain is computationally attractive in the presence of multiple transmitters, whereas it does not use special properties of signals. This paper proposes an improved SDF-based DPD algorithm for strictly noncircular sources. We first derive the property of strictly noncircular signals in the frequency domain. On this basis, the observed frequency-domain vectors at all arrays are concatenated and extended by exploiting the noncircular property, producing extended noise subspaces. Fusing the extended noise subspaces of all frequency components and then performing a unitary transformation, we obtain a cost function for each source location, which is formulated as the smallest eigenvalue of a real-valued matrix. To avoid the exhaustive grid search and solve this nonlinear function efficiently, we devise a Newton-type iterative method using matrix Eigen-perturbation theory. Simulation results demonstrate that the proposed DPD using Newton-type iteration substantially reduces the running time, and its performance is superior to other localization methods for both near-field and far-field noncircular sources.

## 1. Introduction

Noncircular complex signals (e.g., binary-phase-shift-keying (BPSK), multiple-amplitude-shift-keying (MASK) and offset-quadrature-phase-shift-keying (OQPSK) modulated signals) are extensively employed in modern communication systems. During the last few years, the algorithms for noncircular complex sources with application to signal processing [1,2,3,4,5,6,7] have received an upsurge of attention. Different from circular signals, the unconjugated statistical property of noncircular complex signals adds to the amount of available information, which can help to enhance the performance. Since many communication systems offer location-based services, the problem of accurate localization for noncircular sources is worth further investigation. 

Conventional localization methods employ two-step processing [8,9,10,11], where the measurement parameters (e.g., direction of arrival (DOA), time of arrival (TOA) and frequency difference of arrival (FDOA)) are first extracted, and then, the source positions are estimated. In the first step, parameter estimation algorithms for noncircular (NC) signals such as the NC-MUSIC algorithm (multiple signal classification algorithm for noncircular signals) [5] can improve the accuracy, due to the exploitation of the noncircular property. However, the localization algorithm in the second step cannot utilize the properties of signals. Moreover, when multiple sources exist, the two-step localization system confronts the association problem of deciding which of the multiple measurements reported by the observers corresponds to which source. If the measurements are not correctly related to the transmitter, additional errors are produced. As a consequence, these conventional two-step methods are suboptimal and cannot guarantee high localization precision for noncircular sources.

Compared with the conventional two-step methods, direct position determination (DPD) [12,13,14,15,16,17,18,19,20,21,22] is a single-step localization method without computing the intermediate parameters and augments the position estimation with the constraint that all measurements correspond to the same geolocation of the transmitter. Therefore, DPD can avoid the association problem, and its location accuracy has shown to be significantly higher than that of the conventional two-step methods, especially under low signal-to-noise ratio (SNR) conditions [13]. However, the DPD technique often requires the transmission of the sampling data to a central processing location and yields a large amount of computations [16].

Nowadays, DPD algorithms applied to the scenario of widely-separated arrays have been intensively investigated [13,14,15,16,17,18]. A maximum likelihood (ML)-based DPD algorithm was presented in [16], where the locations of multiple sources are decoupled into several lower dimensional optimization problems with the information of uncorrelated waveforms. However, in passive localization systems, signal waveforms are always unknown at the receiver. When solving ML estimators for multiple sources without known waveforms, there is a variety of stray parameters, which requires a substantial computational effort. To this end, Amar and Weiss proposed an iterative algorithm for DPD of multiple unknown signals to reduce the complexity [17]. Although this ML estimator can approach the associated Cramér–Rao bound, its iterative procedure is complicated as it has to perform a grid search during each iteration. 

To make the localization computationally attractive for multiple transmitters, an alternative method is the subspace data fusion (SDF). It has lower computational complexity than ML methods, due to the fact that all source positions are estimated from a MUSIC-like cost function that depends on the parameters of the same dimension as that for only one source. Two SDF-based DPDs were developed in [16] and [18], respectively. One is based on the time domain and implicitly uses the array responses [18], and the other processes the frequency-domain observations and relies on the assumption that the envelopes of the signals are the same at all observers, up to delay and amplitude caused by the propagation channel [16]. Therefore, the latter can exploit the location information embedded in both array responses and TOAs, leading to higher accuracy. However, these SDF-based DPD algorithms were designed for general sources (i.e., circular signals) and did not consider the property of noncircular signals. For this reason, we proposed an SDF-based DPD for strictly noncircular sources observed by a moving array in our early work [19], which exploits the time-domain property of noncircular signals. It can be easily adapted to the scenario of widely-separated arrays. Note that similar to the SDF-based DPD in [18], the DPD developed in our previous work utilizes only the array responses, neglecting the location information in the propagation time between the transmitter and the observer.

In light of the aforementioned related works, in this study, we consider the scenario of widely-separated arrays and assume a line of sight (LOS) propagation of multiple signals with unknown waveforms and unknown complex attenuation at each observer array. The purpose of this study is to develop an efficient SDF-based DPD method in the frequency domain for noncircular sources. The contributions of this paper can be summarized as follows:

- We derive the frequency-domain property of strictly noncircular signals and propose an improved SDF estimator. Based on this noncircular property, we establish an extended frequency-domain observation received by all arrays and compute the extended subspaces, which are implicitly related to array responses and TOAs. Fusing the extended subspaces of all frequency components, a cost function is formulated as the smallest eigenvalue of a symmetric real-valued matrix for each source location, due to a unitary transformation. Therefore, the real-valued eigen-decomposition is required instead of complex computations. Compared with the primitive SDF-based DPDs, this improved SDF estimator retains its superiority for requiring low-dimensional optimization and has higher robustness to noise that comes from exploiting noncircularity.
- We devise a Newton-type iterative algorithm to efficiently solve the prescribed cost function based on matrix Eigen-perturbation theory. It substantially reduces the computations of the straightforward implementation of the optimization for each position, which is always accomplished via a two- or three-dimensional grid search.

The remainder of this paper is organized as follows. Section 2 presents the signal model and formulates the problem. In Section 3, we propose the extended SDF DPD estimator for noncircular sources and devise a Newton-type iterative solution. In Section 4, we show the simulation results and make the discussion. Section 5 concludes the study.

Throughout the paper, upper case and lower case boldface letters will represent matrices and column vectors, respectively. For convenience, we list the notations used in this paper:

${\left\{\cdot \right\}}^{*}$Conjugate.${\left\{\cdot \right\}}^{T}$Transpose.${\left\{\cdot \right\}}^{H}$Conjugate transpose.blkdiag{⋅}Composition of the block diagonal matrix.diag{⋅}Composition of the diagonal matrix.vec{⋅}The “vectorization” operator that turns a matrix into a vector by stacking the columns of the matrix, one below another.⊗Kronecker matrix product.E[⋅]Expectation.tr{⋅}Trace.Re{⋅}Real part.Im{⋅}Imaginary part.${\left[\cdot \right]}_{n}$The *n-*th element of a vector.${\left[\cdot \right]}_{nm}$The *n*,*m-*th entry of a matrix.${\left\|\cdot \right\|}_{2}$Euclidean norm.${\mathbb{C}}^{N\times M}$Set of the N×M complex matrices.${\mathbb{R}}^{N\times M}$Set of the N×M real matrices.

## 2. Problem Formulation

### 2.1. Property of the Noncircular Signal

A signal is normally circular when an arbitrary rotation cannot change its first-order and second-order statistical characteristics, namely rotation invariance. If a signal’s property of rotation invariance is not satisfied, then we take it as a noncircular signal. For a straightforward description, let s(n) be a zero-mean signal, s(n) is considered to be circular with the condition “$E[|s(n){|}^{2}]\neq 0$ and $E[s^{2}(n)]=0$”, whereas s(n) is referred to as being noncircular with the condition “$E[|s(n{)|}^{2}]\neq 0$ and $E[s^{2}(n)]\neq 0$”. Specifically, a strictly noncircular signal can be generated through a phase shift of a real-valued signal [23]. According to this property, we assume s(n) to be a strictly noncircular signal, and therefore, it can be generated as [23]:

(1) $$s(n)=\overset{⌣}{s}(n)e^{i\phi },$$

where $\overset{⌣}{s}(n)$ is a real-valued signal and ϕ signifies the initial phase of s(n). 

Using this time-domain property of a strictly noncircular signal, we can express the discrete Fourier transform (DFT) of s(n) as follows:

(2) $$\begin{array}{ll} \bar{s}(j) & =\sum_{n=0}^{J-1}s(n)\exp\left(-i2\pi nj/J\right)\\ & =\sum_{n=0}^{J-1}\overset{⌣}{s}(n)e^{i\phi }\exp\left(-i2\pi nj/J\right)\;j=0,1,\ldots ,J-1, \end{array}$$

where $\bar{s}(j)$ is the *q-*th DFT coefficient of s(n). The conjugate of $\bar{s}(j)$ is given by:

(3) $${\bar{s}}^{*}(j)=\sum_{n=0}^{J-1}\overset{⌣}{s}(n)e^{-i\phi }\exp\left(i2\pi nj/J\right)\;0,1,\ldots ,J-1.$$

Using exp(i2πnj/J)=exp(−i2πn(J−j)/J), we rewrite (3) by:

(4) $$\begin{array}{ll} {\bar{s}}^{*}(j) & =\sum_{n=0}^{J-1}\overset{⌣}{s}(n)e^{-i\phi }\exp\left(-i2\pi n(J-j)/J\right)\\ & =e^{-i2\phi }\sum_{n=0}^{J-1}\overset{⌣}{s}(n)e^{i\phi }\exp\left(-i2\pi n(J-j)/J\right)\;j=0,1,\ldots ,J-1. \end{array}$$

Based on (2), we have:

(5) $$\bar{s}(J-j)=\sum_{n=0}^{J-1}\overset{⌣}{s}(n)e^{i\phi }\exp\left(-i2\pi n(J-j)/J\right).$$

Combing (4) and (5), it can be checked that:

(6) $${\bar{s}}^{*}(j)=\bar{s}(J-j)e^{-i2\phi }\;j=0,1,\ldots ,J-1.$$

The equation related to the frequency-domain signals as shown in (6) lays the foundation of subsequent theoretical development.

### 2.2. Frequency-Domain Signal Model and Problem Formulation

Let us consider L stationary observers, each of whom is equipped with an antenna array comprising M isotropic sensors. Q transmitters are assumed to radiate uncorrelated narrowband and strictly noncircular signals in the field of interest. We denote $p_{q}\in {\mathbb{R}}^{D\times 1}$ as the position of the q-th transmitter for q=1,2,…,Q. The complex envelope of the outputs of the l-th observer array at time 0≤t≤T is expressed as [16,17]:

(7) $$r_{l}(t)=\sum_{q=1}^{Q}b_{ql}a_{l}(p_{q})s_{q}(t-\tau_{l}(p_{q})-t_{q0})+n_{l}(t)$$

for l=1,2,…,L. Here, $s_{q}(t-\tau_{l}(p_{q})-t_{q0})$ is the complex envelope of the q-th source signal, transmitted at time $t_{q0}$ and delayed by $\tau_{l}(p_{q})$, which indicates the propagation time between the q-th transmitter and the l-th observer. We assume that the source signals are wide-sense stationary. $b_{ql}$ is the unknown complex attenuation coefficient representing the channel effect between the q-th transmitter and the l-th observer. $a_{l}(p_{q})$ is the spatial array response of the l-th observer array to the signal transmitted from the position $p_{q}$, which is related to the corresponding DOA. $n_{l}(t)$ is the white and circularly Gaussian noise mixed through the l-th observer array. The sources and noises are assumed to be uncorrelated and to have a mean of zero.

Similarly to [16], we partition $r_{l}(t)\;(0\leq t\leq T)$ into K sections, and each length equals T/K. Then, sample the signals of each section at time intervals $jT_{s}\left(j=0,1,\ldots ,J-1\right)$ where $T_{s}$ represents the sampling period. Thus, the DFT of the J samples in the k-th section can be expressed as [16]:

(8) $${\bar{r}}_{l}(j,k)=\sum_{q=1}^{Q}b_{ql}a_{l}(p_{q}){\bar{s}}_{q}(j,k)\exp\left(-i2\pi \tau_{l}(p_{q})j/JT_{s}\right)+{\bar{n}}_{l}(j,k)\;j=0,1,\ldots ,J-1,$$

where ${\bar{s}}_{q}(j,k)$ and ${\bar{n}}_{l}(j,k)$ are the j-th DFT coefficients of $s_{q}(t-t_{q0})$ and $n_{l}(t)$ in the k-th section, respectively. For convenience of presentation, we define:

(9) $${\bar{a}}_{l}(j,p_{q})=a_{l}(p_{q})\exp\left(-i2\pi \tau_{l}(p_{q})j/JT_{s}\right),$$

and rewrite ${\bar{r}}_{l}(j,k)$ by:

(10) $${\bar{r}}_{l}(j,k)=\sum_{q=1}^{Q}b_{ql}{\bar{a}}_{l}(j,p_{q}){\bar{s}}_{q}(j,k)+{\bar{n}}_{l}(j,k)\;j=0,1,\ldots ,J-1.$$

We now composite the *j-*th DFT coefficients of all the outputs of L observer arrays and obtain:

(11) $$\begin{array}{ll} \bar{r}(j,k) & ={\left[{\bar{r}}_{1}^{T}(j,k),{\bar{r}}_{2}^{T}(j,k),\ldots ,{\bar{r}}_{L}^{T}(j,k)\right]}^{T}\\ & =\sum_{q=1}^{Q}\Gamma (j,p_{q})b_{q}{\bar{s}}_{q}(j,k)+\bar{n}(j,k)\\ & =\sum_{q=1}^{Q}\alpha_{q}(j){\bar{s}}_{q}(j,k)+\bar{n}(j,k), \end{array}$$

where:

(12) $$\bar{n}(j,k)={\left[{\bar{n}}_{1}^{T}(j,k),{\bar{n}}_{2}^{T}(j,k),\ldots ,{\bar{n}}_{L}^{T}(j,k)\right]}^{T},$$

(13) $$\Gamma (j,p_{q})=\mathrm{blkdiag}\{{\bar{a}}_{1}(j,p_{q}),{\bar{a}}_{2}(j,p_{q}),\ldots ,{\bar{a}}_{L}(j,p_{q})\},$$

(14) $$b_{q}={\left[b_{q1},b_{q2},\ldots ,b_{qL}\right]}^{T},$$

(15) $$\alpha_{q}(j)=\Gamma (j,p_{q})b_{q}.$$

Let us denote $\Phi (j)=\left[\alpha_{1}(j),\alpha_{2}(j),\ldots ,\alpha_{Q}(j)\right]\in {\mathbb{C}}^{LM\times Q}$ and $\bar{s}(j,k)={\left[{\bar{s}}_{1}(j,k),{\bar{s}}_{2}(j,k),\ldots ,{\bar{s}}_{Q}(j,k)\right]}^{T}$. Then, $\bar{r}(j,k)$ can be further expressed in the matrix form as:

(16) $$\bar{r}(j,k)=\Phi (j)\bar{s}(j,k)+\bar{n}(j,k).$$

Therefore, the covariance matrix of $\bar{r}(j,k)$ can be written as:

(17) $$R_{r}(j)=E\left[\bar{r}(j,k){\bar{r}}^{H}(j,k)\right]=\Phi (j)R_{s}(j)\Phi^{H}(j)+\sigma_{n}^{2}I_{LM}.$$

where $R_{s}(j)=E\left[\bar{s}(j,k){\bar{s}}^{H}(j,k)\right]$, $\sigma_{n}^{2}$ denotes the noise power and $I_{LM}$ signifies the LM×LM identity matrix.

Note that Φ(j) is associated with both DOAs and TOAs, and therefore, it contains the location information. The SDF DPD based on the frequency domain [16] relies on the general signal model as shown in (17), which cannot take full advantage of the signal properties. To this end, we come to exploit the noncircularity of signals derived in Section 2.1. 

As the conjugate of $\bar{r}(j,k)$ can be expressed as:

(18) $${\bar{r}}^{*}(j,k)=\Phi^{*}(j){\bar{s}}^{*}(j,k)+{\bar{n}}^{*}(j,k),$$

applying (6) to (18) leads to:

(19) $${\bar{r}}^{*}(j,k)=\Phi^{*}(j)\Delta_{\phi }\bar{s}(J-j,k)+{\bar{n}}^{*}(j,k),$$

where $\Delta_{\phi }=\mathrm{diag}\{e^{-i2\phi_{1}},e^{-i2\phi_{2}},\ldots ,e^{-i2\phi_{Q}}\}$ with $\phi_{q}$ being the initial phase of the *q-*th strictly noncircular source. Replacing j with J−j in (19), it follows that:

(20) $${\bar{r}}^{*}(J-j,k)=\Phi^{*}(J-j)\Delta_{\phi }\bar{s}(j,k)+{\bar{n}}^{*}(J-j,k),$$

for j=0,1,…,J−1. Note that ${\bar{r}}^{*}(J,k)={\bar{r}}^{*}(0,k)$ as the DFT coefficient is periodic with the period J.

By exploiting the unconjugated property of noncircular signals, we combine (16) and (20), and therefore construct the extended observation:

(21) $$\tilde{r}(j,k)={\left[{\bar{r}}^{T}(j,k),{\bar{r}}^{H}(J-j,k)\right]}^{T}=\tilde{\Phi }(j)\bar{s}(j,k)+\tilde{n}(j,k),$$

where $\tilde{n}(j,k)={\left[{\bar{n}}^{T}(j,k),{\bar{n}}^{H}(J-j,k)\right]}^{T}$ and $\tilde{\Phi }(j)={\left[\Phi^{T}(j),\Delta_{\phi }^{T}\Phi^{H}(J-j)\right]}^{T}\in {\mathbb{C}}^{2LM\times Q}$, whose *q-*th column is given by:

(22) $${\tilde{\alpha }}_{q}(j)=\tilde{\Gamma }(j,p_{q}){\tilde{b}}_{q},$$

with:

(23) $$\tilde{\Gamma }(j,p_{q})=\mathrm{blkdiag}\left\{\Gamma (j,p_{q}),\Gamma^{*}(J-j,p_{q})\right\},$$

(24) $${\tilde{b}}_{q}={\left[b_{q}^{T},b_{q}^{H}e^{-i2\phi_{q}}\right]}^{T}.$$

Then, the covariance matrix of the extended observation $\tilde{r}(j,k)$ has the following form:

(25) $$r_{\tilde{r}}(j)=E\left[\tilde{r}(j,k){\tilde{r}}^{H}(j,k)\right]=\tilde{\Phi }(j)R_{s}(j){\tilde{\Phi }}^{H}(j)+\sigma_{n}^{2}I_{2LM}.$$

Until now, we have obtained the frequency-domain data model for our work as shown in (25). It is noteworthy that the dimension of the extended covariance matrix $R_{\tilde{r}}(j)$ is twice that of the covariance matrix $R_{r}(j)$ for circular signals, leading to more available information. To this end, we will apply $R_{\tilde{r}}(j)$ to our problem. The problem that we address now is, given $R_{\tilde{r}}(j)$ for j=0,1,…,J−1, to directly estimate the locations of multiple transmitters without explicitly computing TOAs and DOAs.

## 3. Methods

### 3.1. Extended SDF 

Based on (25), the eigen-decomposition of $R_{\tilde{r}}(j)$ yields:

(26) $$R_{\tilde{r}}(j)=U_{k}^{s}(j)\Sigma_{k}^{s}(j)U_{k}^{\mathrm{sH}}(j)+\sigma_{n}^{2}U_{k}^{n}(j)U_{k}^{\mathrm{nH}}(j),$$

where $\Sigma_{k}^{s}(j)$ is a diagonal matrix with diagonal elements of the Q largest eigenvalues of $R_{\tilde{r}}(j)$. $U_{k}^{s}(j)\in {\mathbb{C}}^{2LM\times Q}$ is the extended signal subspace comprising the eigenvectors corresponding to the Q largest eigenvalues, and it spans the same space as $\tilde{\Phi }(j)$ [5,16,19]. $U_{k}^{n}(j)\in {\mathbb{C}}^{2LM\times (2LM-Q)}$ is the extended noise subspace consisting of the eigenvectors corresponding to the 2LM−Q smallest eigenvalue $\sigma_{n}^{2}$.

According to the subspace orthogonality principle [5,16,19], namely the columns of $\tilde{\Phi }(j)$ are orthogonal to the noise subspace of $R_{\tilde{r}}(j)$, we have:

(27) $${\tilde{\alpha }}_{q}^{H}(j)U_{k}^{n}(j)U_{k}^{\mathrm{nH}}(j){\tilde{\alpha }}_{q}(j)=0\;j=0,1,\ldots ,J-1$$

for q=1,2,…,Q. Fusing J DFT components leads to:

(28) $$\sum_{j=0}^{J-1}{\tilde{\alpha }}_{q}^{H}(j)U_{k}^{n}(j)U_{k}^{\mathrm{nH}}(j){\tilde{\alpha }}_{q}(j)=0.$$

Inserting (22) into (28), it follows that:

(29) $${\tilde{b}}_{q}^{H}Q(p_{q}){\tilde{b}}_{q}=0,$$

where $Q(p_{q})$ is expressed as:

(30) $$Q(p_{q})=\sum_{j=0}^{J-1}{\tilde{\Gamma }}^{H}(j,p_{q})U_{k}^{n}(j)U_{k}^{\mathrm{nH}}(j)\tilde{\Gamma }(j,p_{q})\in {\mathbb{C}}^{2L\times 2L}.$$

It is noteworthy that ${\tilde{b}}_{q}$ in (29) is formulated as ${\tilde{b}}_{q}={\left[b_{q}^{T},b_{q}^{H}e^{-i2\phi_{q}}\right]}^{T}$. Next, we will utilize this structure of ${\tilde{b}}_{q}$ to transform the complex-valued equation in (29) into a real-valued one.

We now rewrite (29) by:

(31) $${\left({\tilde{b}}_{q}e^{i\phi_{q}}\right)}^{H}Q(p_{q}){\tilde{b}}_{q}e^{i\phi_{q}}={{\tilde{b}}^{\prime }}_{q}^{H}Q(p_{q}){{\tilde{b}}^{\prime }}_{q}=0,$$

where ${{\tilde{b}}^{\prime }}_{q}={\tilde{b}}_{q}e^{i\phi_{q}}$. As ${\tilde{b}}_{q}={\left[b_{q}^{T},b_{q}^{H}e^{-i2\phi_{q}}\right]}^{T}$, ${{\tilde{b}}^{\prime }}_{q}$ can be given by:

(32) $${{\tilde{b}}^{\prime }}_{q}={\left[{b^{\prime }}_{q}^{T},{b^{\prime }}_{q}^{H}\right]}^{T}$$

with ${b^{\prime }}_{q}=b_{q}e^{i\phi_{q}}.$ Based on (32), we perform a unitary transformation of ${{\tilde{b}}^{\prime }}_{q}$ to establish a real-valued vector:

(33) $${\overset{⌣}{b}}_{q}=\frac{1}{\sqrt{2}}T{{\tilde{b}}^{\prime }}_{q},$$

where T is the unitary matrix:

(34) $$T=\frac{1}{\sqrt{2}}\left[\begin{array}{cc} I_{L} & I_{L}\\ -iI_{L} & iI_{L} \end{array}\right],$$

and thus, ${\overset{⌣}{b}}_{q}$ can be expressed in the form of (35):

(35) b⌣q=[Re{b′qT},Im{b′qT}]T.

Since $T^{H}T=I_{2L}$, it follows that:

(36) $${{\tilde{b}}^{\prime }}_{q}=\sqrt{2}T^{H}{\overset{⌣}{b}}_{q}.$$

Substituting (36) back into (31), we get:

(37) $${\overset{⌣}{b}}_{q}^{H}Q^{\prime }(p_{q}){\overset{⌣}{b}}_{q}=0,$$

where:

(38) $$Q^{\prime }(p_{q})=TQ(p_{q})T^{H}\in {\mathbb{C}}^{2L\times 2L}.$$

Given that ${\overset{⌣}{b}}_{q}$ is real-valued, we rewrite the left side of the equation in (37) by:

(39)  b⌣qHQ′(pq)b⌣q=b⌣qT(Re{Q′(pq)}+iIm{Q′(pq)})b⌣q=b⌣qTRe{Q′(pq)}b⌣q+ib⌣qTIm{Q′(pq)}b⌣q.

Due to the fact that ${\overset{⌣}{b}}_{q}^{H}Q^{\prime }(p_{q}){\overset{⌣}{b}}_{q}$ is real-valued, we discard the imaginary term, ib⌣qTIm{Q′(pq)}b⌣q, in (39). Then, ${\overset{⌣}{b}}_{q}^{H}Q^{\prime }(p_{q}){\overset{⌣}{b}}_{q}$ is equivalent to:

(40) b⌣qHQ′(pq)b⌣q=b⌣qTRe{Q′(pq)}b⌣q.

According to the above derivation, the complex-valued equation in (29) is transformed into the following real-valued equation:

(41) b⌣qTRe{Q′(pq)}b⌣q=0.

We now replace $U_{k}^{n}(j)$ with ${\hat{U}}_{k}^{n}(j)$, which is computed by the eigen-decomposition of the estimated $R_{\tilde{r}}(j)$:

(42) $${\hat{R}}_{\tilde{r}}(j)=\frac{1}{K}\sum_{k=1}^{K}\tilde{r}(j,k){\tilde{r}}^{H}(j,k).$$

Then, the following optimization model presents the solution for $p_{q}$ and ${\overset{⌣}{b}}_{q}$:

(43) {p^q,b⌣^q}=argminp,b⌣ b⌣TRe{Q′(p)}b⌣.

We observe that the estimation of ${\overset{⌣}{b}}_{q}$ is the eigenvector corresponding to the smallest eigenvalue of Re{Q′(p)}, and the estimation of $p_{q}$ is the point that minimizes the smallest eigenvalue of Re{Q′(p)}. Consequently, the position of the *q-*th transmitter can be obtained by:

(44) $${\hat{p}}_{q}=\underset{p}{\mathrm{argmin}}\;f(p)$$

for q=1,2,…,Q, where:

(45) f(p)=λmin{Re{Q′(p)}}.

Here, $\lambda_{\min}\{\cdot \}$ signifies the smallest eigenvalue of the input matrix. As f(p) is a nonlinear function in terms of the source position, the minimization of f(p) is usually accomplished by a D-dimensional grid search.

### 3.2. Iterative Solution

When the area of interest is large and the grid step size is small, the grid search is computationally demanding. To locate the transmitters quickly, we will devise a Newton-type iterative method to efficiently solve the proposed cost function for each source location. As is known to all, the Newton-type iterative method provides an efficient way to solve nonlinear optimization problems [24]. It requires computing the gradient and Hessian matrix of the cost function, which is quite easy if the cost function is explicitly written. However, the cost function in (45) is the smallest eigenvalue of a symmetric matrix, whose gradient and Hessian matrix cannot be obtained straightforwardly. Therefore, we apply the matrix Eigen-perturbation theory to derive the gradient and Hessian matrix of (45) for each source position. For this purpose, a proposition needs to be introduced first. 

**Proposition:** Assume a symmetric real-valued matrix $X\in {\mathbb{R}}^{N\times N}$ with $\lambda_{\min}=\lambda_{1}\leq \lambda_{2}\leq \cdots \leq \lambda_{N}=\lambda_{\max}$ being its eigenvalues and $e_{1},e_{2},\ldots ,e_{N}$ being the corresponding eigenvectors. Denote δX as the perturbation of X, and hence $\hat{X}=X+\delta X$ where δX and $\hat{X}$ are also symmetric. Then, the eigenvalues of the perturbed matrix $\hat{X}$, ${\hat{\lambda }}_{\min}={\hat{\lambda }}_{1}\leq {\hat{\lambda }}_{2}\leq \cdots \leq {\hat{\lambda }}_{N}={\hat{\lambda }}_{\max}$, can be expressed as [25]:

(46) $${\hat{\lambda }}_{n}=\lambda_{n}+e_{n}^{T}\delta Xe_{n}+e_{n}^{T}\delta XE_{n}\delta Xe_{n}+o\left({\left\|\delta X\right\|}_{2}^{2}\right)\;\left(n=1,2,\ldots ,N\right),$$

where:

(47) $$E_{n}=\sum_{\begin{array}{c} m=1\\ m\neq n \end{array}}^{N}{\left(\lambda_{n}-\lambda_{m}\right)}^{-1}e_{m}e_{m}^{T}\;\left(n=1,2,\ldots ,N\right),$$

and $o\left({\left\|\delta X\right\|}_{2}^{2}\right)$ signifies the infinitesimal term of ${\left\|\delta X\right\|}_{2}^{2}$. This proposition reveals the relationship between the matrix perturbation and the eigenvalues of the disturbed matrix. The proof of (46) can be found in [25].

Now, let us denote X(p)=Re{Q′(p)}, whose eigenvalues and eigenvectors are $\lambda_{1}\leq \lambda_{2}\leq \cdots \leq \lambda_{2L}$ and $e_{1},e_{2},\ldots ,e_{2L}$. Define δp as the perturbation of p and $\hat{p}=p+\delta p$ as the perturbed position. Then, the eigenvalues and eigenvectors of $\hat{X}(\hat{p})$ are ${\hat{\lambda }}_{1}\leq {\hat{\lambda }}_{2}\leq \cdots \leq {\hat{\lambda }}_{2L}$ and ${\hat{e}}_{1},{\hat{e}}_{2},\ldots ,{\hat{e}}_{2L}$, respectively. Denoting $\delta X=\hat{X}(\hat{p})-X(p)$, we obtain the following equation according to the above proposition:

(48) $${\hat{\lambda }}_{1}=\lambda_{1}+e_{1}^{T}\delta Xe_{1}+e_{1}^{T}\delta XE_{1}\delta Xe_{1}+o\left({\left\|\delta X\right\|}_{2}^{2}\right),$$

in which:

(49) $$E_{1}=\sum_{m=2}^{2L}{\left(\lambda_{1}-\lambda_{m}\right)}^{-1}e_{m}e_{m}^{T}.$$

Applying $\mathrm{vec}\{ABC\}=(C^{T}⊗A)\mathrm{vec}\{B\}$ and $\mathrm{tr}\{ABCD\}={\mathrm{vec}}^{T}\{D^{T}\}(C^{T}⊗A)\mathrm{vec}\{B\}$ (see [26]), (48) can be further expressed as a function of δx=vec{δX}:

(50) $$\begin{array}{ll} {\hat{\lambda }}_{1} & =\lambda_{1}+\mathrm{vec}\{e_{1}^{T}\delta Xe_{1}\}+\mathrm{tr}\{e_{1}e_{1}^{T}\delta XE_{1}\delta X\}+o\left({\left\|\delta X\right\|}_{2}^{2}\right)\\ & =\lambda_{1}+(e_{1}^{T}⊗e_{1}^{T})\delta x+\delta x^{T}(E_{1}⊗e_{1}e_{1}^{T})\delta x+o\left({\left\|\delta x\right\|}_{2}^{2}\right). \end{array}$$

where the symmetry of $E_{1}$ and δX is used.

Note that ${\hat{\lambda }}_{1}$ is the cost function of the proposed estimator, and therefore, our purpose is to link δp to ${\hat{\lambda }}_{1}$. As (50) displays the relationship between δx and ${\hat{\lambda }}_{1}$, we proceed to derive the relationship between δx and δp in what follows. 

Denote x(p)=vec{X(p)} and $\hat{x}(\hat{p})=\mathrm{vec}\{\hat{X}(\hat{p})\}$. Based on the result in [27], we can take the second-order Taylor expansion of $\hat{x}(\hat{p})$ around p as:

(51) $$\hat{x}(\hat{p})=x(p)+{\dot{X}}_{p}(p)\delta p+\frac{1}{2}(I_{4L^{2}}⊗\delta p^{T}){\ddot{X}}_{pp}(p)\delta p+o\left({\left\|\delta p\right\|}_{2}^{2}\right),$$

where:

(52) $${\dot{X}}_{p}(p)=\frac{\partial x(p)}{\partial p^{T}}\in {\mathbb{R}}^{4L^{2}\times D}$$

and:

(53) $${\ddot{X}}_{pp}(p)=\left[\begin{array}{c} \frac{\partial^{2}{\left[x(p)\right]}_{1}}{\partial p\partial p^{T}}\\ \frac{\partial^{2}{\left[x(p)\right]}_{2}}{\partial p\partial p^{T}}\\ \vdots \\ \frac{\partial^{2}{\left[x(p)\right]}_{4L^{2}}}{\partial p\partial p^{T}} \end{array}\right]\in {\mathbb{R}}^{4L^{2}D\times D}.$$

For the further derivations of (52) and (53), see Appendix A and Appendix B, respectively.

As $\delta x=\hat{x}(\hat{p})-x(p)$, according to (51), we can relate δx with δp:

(54) $$\delta x={\dot{X}}_{p}(p)\delta p+\frac{1}{2}(I_{4L^{2}}⊗\delta p^{T}){\ddot{X}}_{pp}(p)\delta p+o\left({\left\|\delta p\right\|}_{2}^{2}\right).$$

Then, substituting (54) back into (50) yields:

(55) $$\begin{array}{ll} {\hat{\lambda }}_{1} & =\lambda_{1}+(e_{1}^{T}⊗e_{1}^{T})\delta x+\delta x^{T}(E_{1}⊗e_{1}e_{1}^{T})\delta x+o\left({\left\|\delta x\right\|}_{2}^{2}\right)\\ & =\lambda_{1}+(e_{1}^{T}⊗e_{1}^{T}){\dot{X}}_{p}(p)\delta p+\\ & \;\frac{1}{2}(e_{1}^{T}⊗e_{1}^{T})(I_{4L^{2}}⊗\delta p^{T}){\ddot{X}}_{pp}(p)\delta p+\delta p^{T}{\dot{X}}_{p}^{T}(p)(E_{1}⊗e_{1}e_{1}^{T}){\dot{X}}_{p}(p)\delta p+o\left({\left\|\delta p\right\|}_{2}^{2}\right). \end{array}$$

Because:

(56) $$(e_{1}^{T}⊗e_{1}^{T})(I_{4L^{2}}⊗\delta p^{T}){\ddot{X}}_{pp}(p)\delta p=\delta p^{T}(e_{1}^{T}⊗e_{1}^{T}⊗I_{D}){\ddot{X}}_{pp}(p)\delta p,$$

(55) is equivalent to:

(57) $$\begin{array}{ll} {\hat{\lambda }}_{1} & =\lambda_{1}+(e_{1}^{T}⊗e_{1}^{T}){\dot{X}}_{p}(p)\delta p+\frac{1}{2}\delta p^{T}\left\{\left(e_{1}^{T}⊗e_{1}^{T}⊗I_{D}){\ddot{X}}_{pp}(p)+2{\dot{X}}_{p}^{T}(p)(E_{1}⊗e_{1}e_{1}^{T}){\dot{X}}_{p}(p\right)\right\}\delta p+o\left({\left\|\delta p\right\|}_{2}^{2}\right)\\ & =\lambda_{1}+g^{T}(p)\delta p+\frac{1}{2}\delta p^{T}H(p)\delta p+o\left({\left\|\delta p\right\|}_{2}^{2}\right), \end{array}$$

where g(p) and H(p) are given by:

(58) $$g(p)={\dot{X}}_{p}^{T}(p)(e_{1}⊗e_{1})$$

and:

(59) $$H(p)=(e_{1}^{T}⊗e_{1}^{T}⊗I_{D}){\ddot{X}}_{pp}(p)+2{\dot{X}}_{p}^{T}(p)(E_{1}⊗e_{1}e_{1}^{T}){\dot{X}}_{p}(p).$$

Finally, applying the result in (57) to the objective function in (45), we can express $f(\hat{p})$ in terms of the second-order perturbation of δp as follows:

(60) $$f(\hat{p})=f(p)+g^{T}(p)\delta p+\frac{1}{2}\delta p^{T}H(p)\delta p+o\left({\left\|\delta p\right\|}_{2}^{2}\right).$$

It can be seen from (60) that g(p) and H(p) are the gradient and Hessian matrix of f(p), respectively. Therefore, we can get the estimation of $p_{q}$ following the Newton-type iterative procedure [28]:

(61) $${\hat{p}}_{q}^{\left(i+1\right)}={\hat{p}}_{q}^{\left(i\right)}-\mu^{i}H^{-1}({\hat{p}}_{q}^{\left(i\right)})g({\hat{p}}_{q}^{\left(i\right)}),$$

where the superscript i denotes the iteration number and μ is the step factor deciding the iteration step size. In this way, each transmitter can be located when an initial position is given. Note that the variable step size is used to solve the contradiction between convergence speed and misadjustment, which is usually encountered in the case of fixed step size.

To summarize this iterative process, we provide the procedure below:

**Method: Eigen-Perturbation-Based Newton-Type Iterative Method**1:Set *e* as a positive number that is small enough. Then, choose a suitable $K\in {\mathbb{Z}}^{+}$ and $J\in {\mathbb{Z}}^{+}$ ${\mathbb{Z}}^{+}$ denotes the set of positive integers), and compute the DFT of the observed samples.2:Use (42) to estimate the extended covariance matrix ${\hat{R}}_{\tilde{r}}(j)$ for j=0,1,…,J−1..3:Compute the eigenvectors corresponding to the 2LM−Q smallest eigenvalues of ${\hat{R}}_{\tilde{r}}(j)$ for j=0,1,…,J−1 and thus obtain the extended noise subspaces ${\hat{U}}_{k}^{n}(j)$ for j=0,1,…,J−1.4:**for** *q* = 1,2,…,*Q* **do**5:Initialize ${\hat{p}}_{q}^{\left(0\right)}$, and make i←0.6:Insert ${\hat{p}}_{q}^{\left(i\right)}$ into (58) and (59) to compute $g({\hat{p}}_{q}^{\left(i\right)})$ and $H({\hat{p}}_{q}^{\left(i\right)})$.7:Compute $\Delta_{i}={\left\|g({\hat{p}}_{q}^{\left(i\right)})\right\|}_{2}$. If $\Delta_{i}\leq e,\;{\hat{p}}_{q}^{\left(i\right)}$ is the estimated source position, and stop iterating; otherwise, continue.8:Use (61) to update ${\hat{p}}_{q}^{\left(i+1\right)}$. Make i←i+1, and go to Step 6.9: **end for**

**Remark 1.** *Similarly to existing SDF-based DPDs, the method proposed above has an advantage over ML-based methods in the presence of multiple transmitters as it estimates the positions in a decoupled manner. Different from ML functions, which have only one global extremum, our cost function exhibits a few minima at the estimated source positions. Considering that the Newton-type iteration is a local search method and is bound to converge to a local minimum, we use it as a fast converging search procedure instead of the fine search. The initial guess will determine the position to which source the converging local minimum corresponds. Therefore, our iterative method for solving the proposed cost function relies on the initialization to resolve multiple sources, whereas the optimization methods for solving the ML functions require a good initial guess to avoid the local minima. For the initialization of our iterative method, the initial position of each transmitter can be effectively obtained through a coarse search as long as it can separate multiple sources. This can guarantee the accurate and fast localization.*

### 3.3. Computational Complexity

The main computational costs of the proposed methods using exhaustive grid search and using Newton-type iteration and the SDF DPD method for circular sources based on the frequency domain [16] include four parts, i.e., computing the DFT, estimating the covariance matrix, performing the eigen-decomposition and solving the cost function. Table 1 lists the order of the number of real-valued multiply operations in each part consumed by the aforementioned methods, where $N_{p}$ denotes the number of position grid points and $N_{iter}$ represents the iteration number required for convergence. 

As shown in Table 1, the SDF-based DPD in [16] is more computationally efficient than the proposed DPD using the exhaustive search because the dimension of the covariance matrix employed in our algorithm is twice as large as the dimension of the covariance matrix used in [16]. On the other hand, the complexity of the proposed DPD using Newton-type iteration is much lower than that of the exhaustive search implementation since $N_{p}$ is usually much larger than $N_{iter}$. For further comparison, we will examine their computer running times through the simulations described in Section 4.

## 4. Results

The purpose of this section is to present the simulation results and performance analysis relying on Monte Carlo simulations. We consider the scenario of three observers located at the positions [3,3]${}^{T}(\mathrm{km})$, [3,−3]${}^{T}(\mathrm{km})$ and ${\left[-3,3\right]}^{T}(\mathrm{km})$. Each observer is equipped with a uniform linear array (ULA) composed of M=3 sensors. The adjacent sensors are spaced with 0.5λ, where λ denotes the wavelength. Without loss of generality, we assume two transmitters in the field of interest. They arrive at observers with attenuation coefficient vectors being $b_{1}=$ [10.94 + 0.34i,0.77 + 0.64i]^T^ and $b_{2}=$ [10.87 + 0.5i,0.77 + 0.64i]^T^, respectively. The baseband signal waveforms are generated as narrowband strictly noncircular signals with identical power $\sigma_{s}^{2}$, whose initial phases are $\phi_{1}=\pi /5(\mathrm{rad})$ and $\phi_{2}=\pi /3(\mathrm{rad})$. The noises are uncorrelated, circularly symmetric Gaussian random variables with constant power $\sigma_{n}^{2}$. Unless stated otherwise, each observer collects signals in K=50 sections each of J=16 frequencies using a sample rate of 40k (samples/s). For comparison, we invoke the following five estimators:

- Proposed DPD estimator using the devised Newton-type iterative method.
- Proposed DPD estimator using the exhaustive search.
- SDF-based DPD estimator for general circular sources in the frequency domain [16] (denoted by FD-DPD).
- SDF-based DPD estimator for noncircular sources in time domain [19] (denoted by NC TD-DPD).
- Two-step processing estimator: DOA estimation using the NC-MUSIC algorithm [5] and TOA estimation using the ML criterion for noncircular signals [7] at each observer, and pseudo-linear weighted least square localization with the DOA and TOA estimates from all observers used as the data, where DOA and TOA estimates are assumed to be associated with the correct transmitter (denoted by two-step).

In the simulations, the exhaustive search of DPD estimators is implemented through a coarse search with a 0.01-km resolution and then a fine search with a 0.001-km resolution. For the Newton-type iterative method, the positions are initialized using a coarse search with a 0.1-km resolution, which is capable of resolving sources, as will be shown later on. The step factor is μ=0.9. Under different experimental conditions, each simulation is conducted in 1000 Monte Carlo trials, and the location root mean square error (RMSE) is used to assess the localization performance:

(62) $${\mathrm{RMSE}}_{q}=\sqrt{\frac{1}{1000}\sum_{n=1}^{1000}{\left\|p_{q}-{\hat{p}}_{n,q}\right\|}^{2}}$$

for the *q-*th source, where ${\hat{p}}_{n,q}^{}$ denotes the estimation of $p_{q}$ in the *n-*th Monte Carlo trial. 

**Figure 1:**
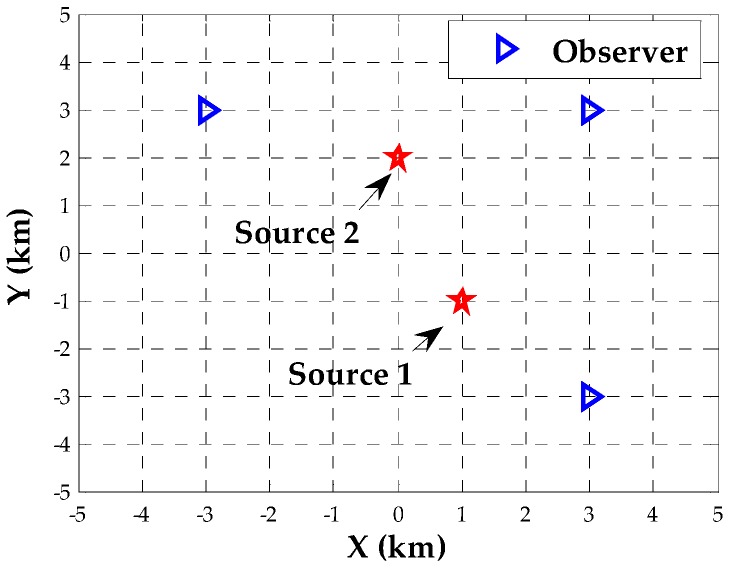
Scenario of three observers and two near-field sources placed on the ground.

**Figure 2:**
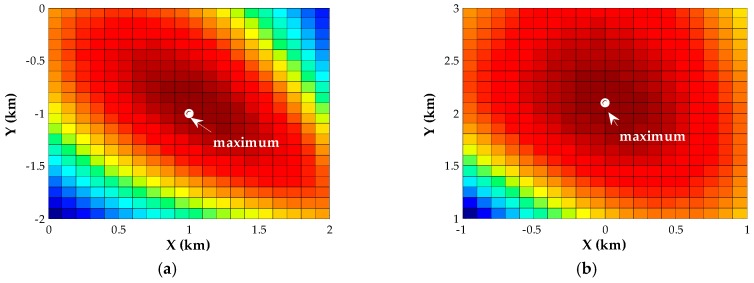
Evaluation of the inverse cost function over the area around the true positions of near-field sources, where different colors represent different magnitudes of amplitude. (**a**) Source 1; (**b**) Source 2.

**Figure 3:**
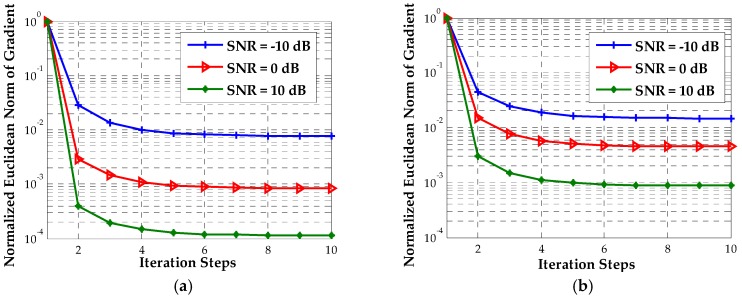
The normalized Euclidean norm of gradient versus iteration steps. (**a**) Source 1; (**b**) Source 2.

This section will show the performance of the proposed algorithms in seven examples. We begin with an analysis of the initial condition and the convergence performance of our Newton-type iterative method. Two near-field sources are assumed to be located at $p_{1}={\left[1,-1\right]}^{T}\;(\mathrm{km})$ and $p_{2}={\left[0,2\right]}^{T}\;(\mathrm{km})$, and the corresponding scenario is shown in Figure 1. For different SNRs, we assess the inverse cost function of the proposed estimator over the candidate positions with a 0.1-km resolution. Figure 2 displays the corresponding evaluation of the inverse cost function within a square area of 2 × 2 (km × km) around the true positions of the two transmitters at SNR = 0 dB. It can be seen that the maximum of the inverse cost function appears near the true position of each source, and there is no pseudo peak in this area, which indicates that the coarse search with a 0.1-km resolution can provide the effective initial positions. Then, we evaluate the normalized Euclidean norm of the gradient for each source versus the number of iteration steps, when the SNR is −10 dB, 0 dB and 10 dB, respectively. As shown in Figure 3, fewer than six iterations are required for convergence in the whole range of SNRs.

In the second example, for the simulation conditions given above, we roughly evaluate the computer running time of each of the prescribed location algorithms based on an average of 1000 estimates, when the SNR is 0 dB. The computer machine used is a ThinkPad laptop equipped with a 2.50-GHz Intel Core CPU and 8 GB RAM. The results are listed in Table 2. To make our results as comprehensive as possible, we also implement the fine search through the Nelder–Mead simplex search approach [29], which is a kind of local search method, and compare its average running time with that of the proposed Newton-type iterative method. We note that the complexity of our algorithm using the exhaustive grid search is larger than those of other approaches. As expected, our iterative method is the most computationally attractive among the six algorithms, and it accomplishes the location in about two thirds of the time required for the Nelder–Mead simplex search. Compared with the exhaustive search implementation, the running time of our iterative method is reduced by approximately ten times. This result agrees with the analysis in Section 3.3.

**Figure 4:**
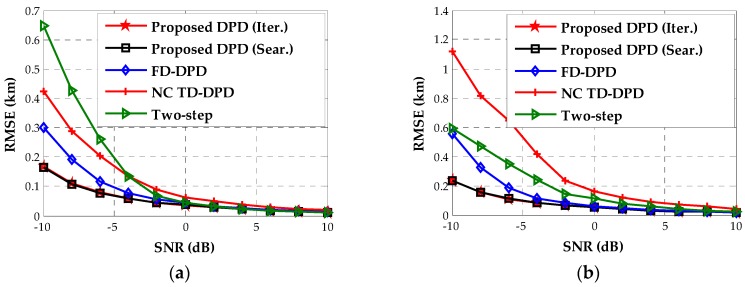
The estimated RMSEs versus SNR for near-field sources. (**a**) Source 1; (**b**) Source 2.

The third example will assess the accuracy of our algorithm for the near-field sources as shown in Figure 1. We plot the RMSE curves of the proposed DPD algorithms and other positioning algorithms versus SNR in Figure 4. It is obvious that the proposed DPD algorithms significantly outperform other comparison algorithms at low SNRs. As the SDF-based DPD in the time domain exploits only the DOA information, it exhibits the lowest accuracy even when the SNR is sufficiently high. These results indicate that both the noncircularity and the TOA information are helpful for enhancing accuracy. Furthermore, since the proposed DPD methods and two-step approach utilize the location information embedded in DOAs and TOAs for noncircular sources, the improved localization performance with respect to the traditional two-step method reveals the advantage of the DPD technique. As the RMSE curves of the proposed estimator using the Newton-type iterative method and that using Nelder–Mead simplex search overlap with each other, we provide their RMSE values in Table 3. It can be observed that the difference of the accuracy between two methods is not more than 0.001 km. 

**Figure 5:**
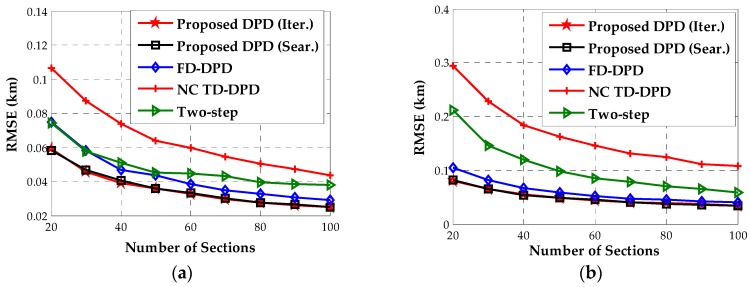
The estimated RMSEs versus the number of sections for near-field sources. (**a**) Source 1; (**b**) Source 2.

In the fourth example, we simulate the location performance with the number of sections K varying from 20 to 100 when the SNR is 0 dB. The location geometry corresponds to that in the foregoing examples. As shown in Figure 5, the performance of our algorithm remains promising in the range of different numbers of sections. When the sample number becomes smaller, this performance improvement turns out to be more prominent.

**Figure 6:**
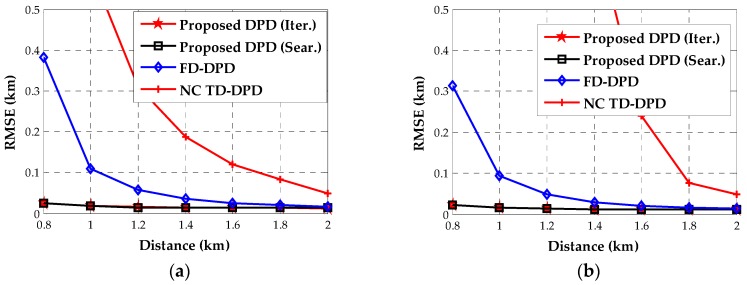
The estimated RMSEs for different distances of near-field sources. (**a**) Source 1; (**b**) Source 2.

In the fifth example, we study the location accuracy when the distance between the two transmitters changes. The two transmitters are assumed to be placed at $p_{1}={\left[0,-d/2\right]}^{T}\;(\mathrm{km})$ and $p_{2}={\left[0,d/2\right]}^{T}\;(\mathrm{km})$, respectively. The SNR is as high as 10 dB. As d increases from 0.8 km to 2 km, we depict the RMSEs of the proposed DPDs and the existing SDF-based DPDs. Since the two-step approach can hardly separate the two transmitters with small distances, we do not show its RMSE curves. It can be seen from Figure 6 that our algorithms performs considerably better than other DPDs when the two sources are closely located. Specifically, the SDF-based DPD in the time domain fails to localize the second transmitter in the case of d<1.6(km). This demonstrates that our algorithm exhibits superior resolution compared to the existing SDF-based DPDs.

**Figure 7:**
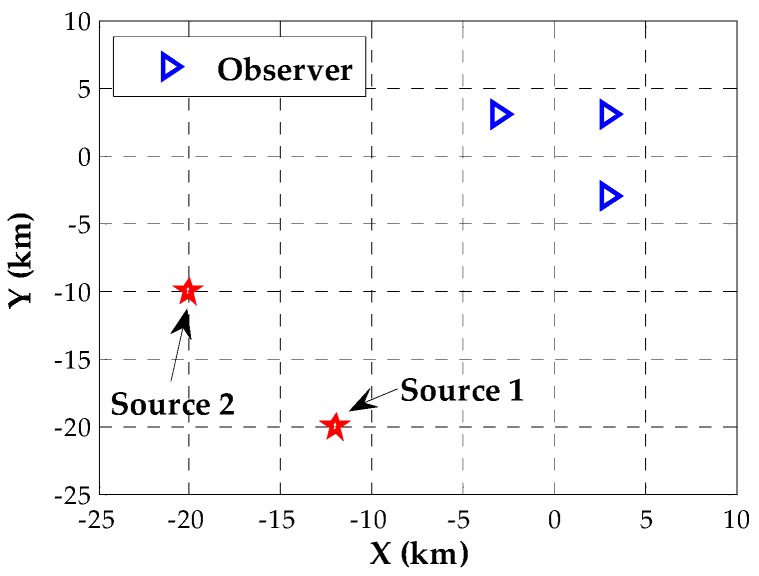
Scenario of three observers and two far-field sources placed on the ground.

**Figure 8:**
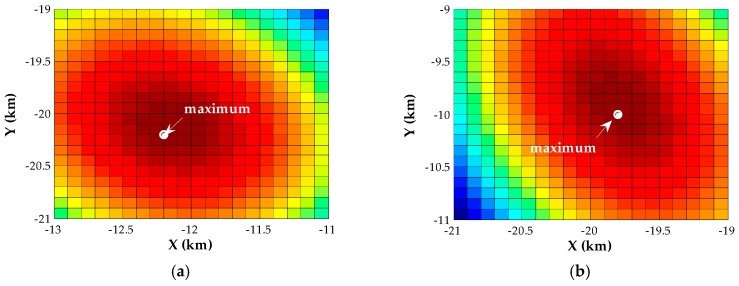
Evaluation of the inverse cost function over the area around the true positions of far-field sources, where different colors represent different magnitudes of amplitude. (**a**) Source 1; (**b**) Source 2.

In this example, we consider two far-field transmitters located at $p_{1}={\left[-12,-20\right]}^{T}(\mathrm{km})$ and $p_{2}={\left[-20,-10\right]}^{T}(\mathrm{km})$ as illustrated in Figure 7. To examine the initial positions of the far-field sources obtained by the coarse search with a 0.1-km resolution, we compute the inverse cost function of the proposed estimator over the candidate positions with a grid step size of 0.1 km for different SNRs. The results for SNR = 0 dB are shown in Figure 8. We note that the resolution of this coarse search is sufficiently high to resolve the far-field sources and thus to obtain the effective initialization.

**Figure 9:**
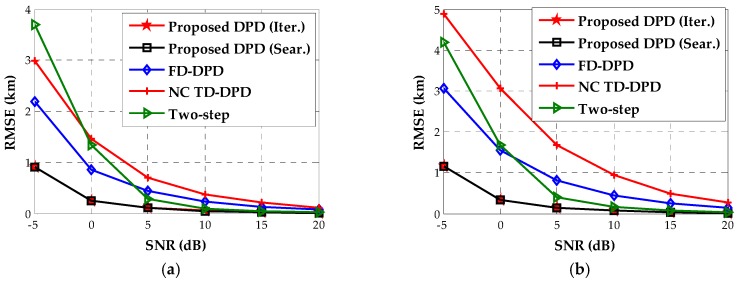
The estimated RMSEs versus SNR for far-field sources. (**a**) Source 1; (**b**) Source 2.

Finally, we come to examine the running time and location accuracy of the proposed algorithms for the far-field sources as shown in Figure 7. When the SNR is 0 dB, we evaluate the average running time of each location algorithm. The corresponding results are shown in Table 4, which confirms that the devised iterative method is less complex than other methods. Especially, in contrast to the implementation of the Nelder–Mead simplex search, our Newton-type iterative method serves as a faster local search procedure. Under the same geometry, Figure 9 displays the location accuracy for the two far-field sources versus SNR, indicating that the proposed algorithms still hold the lowest RMSEs. When the SNR is below 0 dB, the RMSEs of the proposed method are much lower than those of other location algorithms by at least 0.5 km. Moreover, we notice that the performance of the two-step approach (where the DOA and TOA estimations are designed for noncircular signals in the first step) is severely degraded at low SNRs, whereas it performs better than the SDF-based DPD in the frequency domain when the SNR is higher than 5 dB. Comparing this result with that in Figure 4, we find that the exploitation of the noncircular property is more crucial to the performance enhancement for the far-field sources than for the near-field sources. Besides, we show the RMSEs of the proposed estimators using the Newton-type iterative method and using the Nelder–Mead simplex search in Table 5. It can be seen that the accuracies of these two local search methods have no significant difference for the far-field sources.

Additionally, the results indicate that the location accuracy of the proposed DPD using the Newton-type iterative method is approximately the same as those of the Nelder–Mead simplex search and the exhaustive grid search. Considering that the running time of the Newton-type iterative method is much shorter than those of other methods (Table 2 and Table 4), this iterative method provides an efficient way to solve our DPD problem.

## 5. Conclusions

This study has investigated an efficient single-step localization method for noncircular sources received by widely-separated arrays. Through deriving and exploiting the potential of strictly noncircular signals in the frequency domain, the proposed algorithm fuses all the extended noise subspaces of all frequency components for the locations of multiple noncircular sources directly without estimating DOAs and TOAs. Relying on a unitary transformation, we have derived a cost function for each transmitter position, which is formulated as the smallest eigenvalue of a symmetric real-valued matrix. To further reduce the computation load of the exhaustive search for solving this cost function, we have devised a Newton-type iterative method based on the matrix Eigen-perturbation theory. Note that this iterative method can be easily extended to solve any estimator whose cost function is the eigenvalue of a specified matrix. Simulation results demonstrate that the proposed location method using Newton-type iteration is computationally efficient. Its running time is approximately one tenth of that of the exhaustive grid search and is nearly two thirds of the running time of the Nelder–Mead simplex search. Furthermore, our method shows greater performance robustness at low SNRs and under poor location geometry, compared with the conventional two-step approach and existing SDF DPD algorithms. Specifically, when the SNR is below 0 dB, the RMSEs of the proposed method are much lower than those of the existing SDF DPDs by at least 0.5 km for the simulated far-field sources.

To further improve the DPD performance and obtain the optimal location accurately and quickly, it is a possible direction to design a hybrid method combining an evolutionary algorithm and a fast local search approach for solving the ML-based DPD problem.

## Figures and Tables

**Table 1:** Computational complexity.

Method	Complexity				Comments
	Computing DFT	Estimating Covariance Matrix	Eigen-Decomposition	Solving Cost Function	
SDF-based DPD in [16]	$O\left(2LMKJ\log_{2}J\right)$	$O\left(4L^{2}M^{2}KJ\right)$	$O\left(4L^{3}M^{3}J\right)$	$O\left((4L^{2}M^{2}QJ+6L^{2}M^{2}J+8L^{3})QN_{p}\right)$	- For Q circular sources
Proposed DPD using exhaustive search	$O\left(2LMKJ\log_{2}J\right)$	$O\left(16L^{2}M^{2}KJ\right)$	$O\left(32L^{3}M^{3}J\right)$	$O\left(\left(\begin{array}{l} 8L^{3}(M^{2}+M)J-\\ 8L^{2}(M+1)QJ+8L^{3} \end{array}\right)QN_{p}\right)$	- For Q strictly noncircular sources
Proposed DPD using Newton-type iteration	$O\left(2LMKJ\log_{2}J\right)$	$O\left(16L^{2}M^{2}KJ\right)$	$O\left(32L^{3}M^{3}J\right)$	$O\left(\left(\begin{array}{l} (8L^{3}(M^{2}+M)J-\\ 8L^{2}(M+1)QJ){\left(D+1\right)}^{2}+\\ 4L^{2}D^{2}+8L^{3}+4L^{2}D+\\ 4L^{}D^{2}+D^{3}+2LD \end{array}\right)QN_{iter}\right)$	- For Q strictly noncircular sources - Complexity for initialization is not included

**Table 2:** Average runtime for near-field sources. FD, frequency domain; TD, time domain; NC, noncircular.

Method	Runtime (s)
Proposed DPD (Newton-type Iterative Method)	0.0818
Proposed DPD (Exhaustive Grid Search)	0.7802
Proposed DPD (Nelder–Mead Simplex Search)	0.1231
FD-DPD	0.5274
NC TD-DPD	0.5070
Two-step	0.3120

**Table 3:** The estimated RMSEs of the proposed DPDs using the Newton-type iterative method and Nelder–Mead simplex search for near-field sources (km).

Source	Method	SNR (dB)					
		−10	−6	−2	2	6	10
Source 1	Newton-type Iterative Method	0.167	0.079	0.049	0.028	0.017	0.011
	Nelder-Mead Simplex Search	0.166	0.078	0.050	0.029	0.017	0.012
Source 2	Newton-type Iterative Method	0.257	0.109	0.063	0.038	0.024	0.015
	Nelder–Mead Simplex Search	0.258	0.110	0.063	0.039	0.023	0.016

**Table 4:** Average runtime for far-field sources.

Method	Runtime (s)
Proposed DPD (Newton-type Iterative Method)	0.0856
Proposed DPD (Exhaustive Grid Search)	0.7869
Proposed DPD (Nelder–Mead Simplex Search)	0.1330
FD-DPD	0.5144
NC TD-DPD	0.5072
Two-step	0.3132

**Table 5:** The estimated RMSEs of the proposed DPDs using the Newton-type iterative method and Nelder–Mead simplex search for far-field sources (km).

Source	Method	SNR (dB)					
		−5	0	5	10	15	20
Source 1	Newton-type Iterative Method	0.935	0.243	0.108	0.053	0.029	0.016
	Nelder-Mead Simplex Search	0.933	0.250	0.101	0.050	0.029	0.016
Source 2	Newton-type Iterative Method	1.168	0.325	0.135	0.068	0.035	0.020
	Nelder–Mead Simplex Search	1.160	0.324	0.141	0.068	0.036	0.021

## Appendix A

Derivation of the expression for ${\dot{X}}_{p}(p)$:

According to the definition of ${\dot{X}}_{p}(p)$, it is computed by:

(A1) X˙p(p)=∂x(p)∂pT=∂vec{X(p)}∂pT=∂vec{Re{Q′(p)}}∂pT.

For convenience, we restate the expression for $Q^{\prime }(p)$ here:

(A2) $$Q^{\prime }(p)=\sum_{j=0}^{J-1}T{\tilde{\Gamma }}^{H}(j,p)U_{k}^{n}(j)U_{k}^{\mathrm{nH}}(j)\tilde{\Gamma }(j,p)T^{H}.$$

As the operations Re{⋅}, vec{⋅} and taking the derivative can be processed out of order, as well as this ends up with the same result, we first come to derive $\frac{\partial \mathrm{vec}\{Q^{\prime }(p)\}}{\partial p^{T}}$.

Based on (A2) and using $\mathrm{vec}\{ABC\}=(C^{T}⊗A)\mathrm{vec}\{B\}$ (see [26]), we can express $\mathrm{vec}\{Q^{\prime }(p)\}$ as:

(A3) $$\mathrm{vec}\{Q^{\prime }(p)\}=\sum_{j=0}^{J-1}\left(T^{*}⊗\left(T{\tilde{\Gamma }}^{H}(j,p)U_{k}^{n}(j)U_{k}^{\mathrm{nH}}(j)\right)\right)\mathrm{vec}\{\tilde{\Gamma }(j,p)\},$$

and it can be also written as:

(A4) $$\begin{array}{ll} \mathrm{vec}\{Q^{\prime }(p)\} & =\sum_{j=0}^{J-1}\left(\left(T^{*}{\tilde{\Gamma }}^{T}(j,p)U_{k}^{n*}(j)U_{k}^{n}{}^{T}(j)\right)⊗T\right)\mathrm{vec}\{{\tilde{\Gamma }}^{H}(j,p)\}\\ & =\sum_{j=0}^{J-1}\left(\left(T^{*}{\tilde{\Gamma }}^{T}(j,p)U_{k}^{n*}(j)U_{k}^{n}{}^{T}(j)\right)⊗T\right)P\mathrm{vec}\{{\tilde{\Gamma }}^{*}(j,p)\}, \end{array}$$

where P is a vec-permutation matrix that transforms $\mathrm{vec}\{{\tilde{\Gamma }}^{*}(j,p)\}$ to $\mathrm{vec}\{{\tilde{\Gamma }}^{H}(j,p)\}$, i.e., $\mathrm{vec}\{{\tilde{\Gamma }}^{H}(j,p)\}=P\mathrm{vec}\{{\tilde{\Gamma }}^{*}(j,p)\}$.

Define:

(A5) $${\dot{\tilde{\Gamma }}}_{p}(j,p)=\frac{\partial \mathrm{vec}\{\tilde{\Gamma }(j,p)\}}{\partial p^{T}}.$$

Then, combing the results in (A3) and (A4), we can calculate the gradient of $\mathrm{vec}\{Q^{\prime }(p)\}$ by:

(A6) $$\frac{\partial \mathrm{vec}\{Q^{\prime }(p)\}}{\partial p^{T}}=w_{1}(p)+w_{2}(p),$$

where:

(A7) $$w_{1}(p)=\sum_{j=0}^{J-1}\left(T^{*}⊗\left(T{\tilde{\Gamma }}^{H}(j,p)U_{k}^{n}(j)U_{k}^{\mathrm{nH}}(j)\right)\right){\dot{\tilde{\Gamma }}}_{p}(j,p)$$

and:

(A8) $$w_{2}(p)=\left(\left(T^{*}{\tilde{\Gamma }}^{T}(j,p)U_{k}^{n*}(j)U_{k}^{\mathrm{nT}}(j)\right)⊗T\right)P{\dot{\tilde{\Gamma }}}_{p}^{*}(j,p).$$

Finally, extracting the real part of (A6), we obtain:

(A9) X˙p(p)=Re{∂vec{Q′(p)}∂pT}=Re{w1(p)+w2(p)},

which completes our derivation.

## Appendix B

Derivation of the expression for ${\ddot{X}}_{pp}(p)$:

To obtain the explicit expression for ${\ddot{X}}_{pp}(p)$, we first derive each D×D sub-block as shown in (53), i.e.,

(A10) ∂2[x(p)]2mL−2L+n∂p∂pT=∂2Re{[Q′(p)]n,m}∂p∂pT=Re{∂2[Q′(p)]n,m∂p∂pT},

where n,m=1,2,…,2L.

Let $v_{n}$ be the 2L×1 unit vector with the *n*-th element being one, and $v_{m}$ is defined similarly as $v_{n}$. Therefore, the *n*,*m-*th entry of $Q^{\prime }(p)$ is given by:

(A11) $${\left[Q^{\prime }(p)\right]}_{n,m}=\sum_{j=0}^{J-1}v_{n}^{T}T{\tilde{\Gamma }}^{H}(j,p)U_{k}^{n}(j)U_{k}^{\mathrm{nH}}(j)\tilde{\Gamma }(j,p)T^{H}v_{m}.$$

Denote $\dot{\tilde{\Gamma }}{}^{d}(j,p)=\frac{\partial \tilde{\Gamma }(j,p)}{\partial p_{d}}$ and $\ddot{\tilde{\Gamma }}{}_{d_{1}d_{2}}(j,p)=\frac{\partial^{2}\tilde{\Gamma }(j,p)}{\partial p_{d_{1}}\partial p_{d_{2}}}$, where $p_{d}$ is the *d-*th element of p. Using $\mathrm{tr}\{ABCD\}={\mathrm{vec}}^{T}\{D^{T}\}(C^{T}⊗A)\mathrm{vec}\{B\}$ and $\mathrm{tr}\{ABCD\}={\mathrm{vec}}^{T}\{D\}(A⊗C^{T})\mathrm{vec}\{B^{T}\}$ (see [26]), we can compute the second-order derivative of ${\left[Q^{\prime }(p)\right]}_{n,m}$ as follows:

(A12) $$\begin{array}{l} \frac{\partial^{2}{\left[Q^{\prime }(p)\right]}_{n,m}}{\partial p_{d1}\partial p_{d2}}\\ =\sum_{j=0}^{J-1}\left\{\begin{array}{l} v_{n}^{T}T{\ddot{\tilde{\Gamma }}}_{d_{1}d_{2}}^{H}(j,p)U_{k}^{n}(j)U_{k}^{n}{}^{H}(j)\tilde{\Gamma }(j,p)T^{H}v_{m}+v_{n}^{T}T{\dot{\tilde{\Gamma }}}_{d_{1}}^{H}(j,p)U_{k}^{n}(j)U_{k}^{\mathrm{nH}}(j){\dot{\tilde{\Gamma }}}_{d_{2}}(j,p)T^{H}v_{m}+\\ v_{n}^{T}T{\dot{\tilde{\Gamma }}}_{d_{2}}^{H}(j,p)U_{k}^{n}(j)U_{k}^{\mathrm{nH}}(j)\dot{\tilde{\Gamma }}{}^{d_{1}}(j,p)T^{H}v_{m}^{}+v_{n}^{T}T{\tilde{\Gamma }}^{H}(j,p)U_{k}^{n}(j)U_{k}^{n}{}^{H}(j)\ddot{\tilde{\Gamma }}{}_{d_{2}d_{1}}(j,p)T^{H}v_{m} \end{array}\right\}\\ =\sum_{j=0}^{J-1}\left\{\begin{array}{l} v_{n}^{T}T{\ddot{\tilde{\Gamma }}}_{{}_{d_{1}d_{2}}}^{H}(j,p)U_{k}^{n}(j)U_{k}^{n}{}^{H}(j)\tilde{\Gamma }(j,p)T^{H}v_{m}^{}+\mathrm{tr}\{U_{k}^{n}(j)U_{k}^{\mathrm{nH}}(j)\dot{\tilde{\Gamma }}{}_{d_{2}}(j,p)T^{H}v_{m}v_{n}^{T}T{\dot{\tilde{\Gamma }}}_{d_{1}}^{H}(j,p)\}+\\ \mathrm{tr}\{T^{H}v_{m}^{}v_{n}^{T}T{\dot{\tilde{\Gamma }}}_{d_{2}}^{H}(j,p)U_{k}^{n}(j)U_{k}^{n}{}^{H}(j){\dot{\tilde{\Gamma }}}_{d_{1}}(j,p)\}+v_{n}^{T}T{\tilde{\Gamma }}^{H}(j,p)U_{k}^{n}(j)U_{k}^{\mathrm{nH}}(j)\ddot{\tilde{\Gamma }}{}_{d_{2}d_{1}}(j,p)T^{H}v_{m} \end{array}\right\}\\ =\sum_{j=0}^{J-1}\left\{\begin{array}{l} v_{m}^{T}T^{*}{\tilde{\Gamma }}^{T}(j,p)U_{k}^{n*}(j)U_{k}^{n}{}^{T}(j){\ddot{\tilde{\Gamma }}}_{d_{1}d_{2}}^{*}(j,p)T^{T}v_{n}+\\ {\mathrm{vec}}^{T}\{{\dot{\tilde{\Gamma }}}_{d_{1}}^{*}(j,p)\}\left(\left(T^{T}v_{n}^{}v_{m}^{T}T^{*}\right)⊗\left(U_{k}^{n}(j)U_{k}^{\mathrm{nH}}(j)\right)\right)\mathrm{vec}\{{\dot{\tilde{\Gamma }}}_{d_{2}}(j,p)\}+\\ {\mathrm{vec}}^{T}\{{\dot{\tilde{\Gamma }}}_{d_{1}}(j,p)\}\left(\left(T^{H}v_{m}^{}v_{n}^{T}T\right)⊗\left(U_{k}^{n*}(j)U_{k}^{n}{}^{T}(j)\right)\right)\mathrm{vec}\{{\dot{\tilde{\Gamma }}}_{d_{2}}^{*}(j,p)\}+\\ v_{n}^{T}T{\tilde{\Gamma }}^{H}(j,p)U_{k}^{n}(j)U_{k}^{n}{}^{H}(j)\ddot{\tilde{\Gamma }}{}_{d_{1}d_{2}}(j,p)T^{H}v_{m}^{} \end{array}\right\} \end{array}$$

for $d_{1},d_{2}=1,2,\ldots ,D$. Note that the terms on the right side of (A12) are the $d_{1},d_{2}$-th entries of:

(A13) $$W_{1}(p)=\sum_{j=0}^{J-1}\left(I_{D}⊗\left(v_{m}^{T}T^{*}{\tilde{\Gamma }}^{T}(j,p)U_{k}^{n*}(j)U_{k}^{n}{}^{T}(j)\right)\right){\ddot{\tilde{\Gamma }}}_{{}_{pp}}^{*}(j,p)\left(I_{D}⊗T^{T}v_{n}\right),$$

(A14) $$W_{2}(p)=\sum_{j=0}^{J-1}{\dot{\tilde{\Gamma }}}_{p}^{H}(j,p)\left(\left(T^{T}v_{n}v_{m}^{T}T^{*}\right)⊗\left(U_{k}^{n}(j)U_{k}^{\mathrm{nH}}(j)\right)\right){\dot{\tilde{\Gamma }}}_{{}^{p}}^{}(j,p),$$

(A15) $$W_{2}^{T}(p)=\sum_{j=0}^{J-1}{\dot{\tilde{\Gamma }}}_{p}^{T}(j,p)\left(\left(T^{H}v_{m}v_{n}^{T}T\right)⊗\left(U_{k}^{n*}(j)U_{k}^{n}{}^{T}(j)\right)\right){\dot{\tilde{\Gamma }}}_{p}^{*}(j,p),$$

and:

(A16) $$W_{3}(p)=\sum_{j=0}^{J-1}\left(I_{D}⊗\left(v_{n}^{T}T{\tilde{\Gamma }}^{H}(j,p)U_{k}^{n}(j)U_{k}^{n}{}^{H}(j)\right)\right)\ddot{\tilde{\Gamma }}{}_{pp}(j,p)\left(I_{D}⊗T^{H}v_{m}^{}\right),$$

respectively, where $\dot{\tilde{\Gamma }}{}_{p}(j,p)$ has been defined in (A5), and $\ddot{\tilde{\Gamma }}{}_{pp}(j,p)$ has the following structure:

(A17) $$\ddot{\tilde{\Gamma }}{}_{pp}(j,p)=\left[\begin{array}{ccc} \ddot{\tilde{\Gamma }}{}_{11}(j,p) & \cdots & \ddot{\tilde{\Gamma }}{}_{1D}(j,p)\\ \vdots & \ddots & \vdots \\ \ddot{\tilde{\Gamma }}{}_{D1}(j,p) & \cdots & \ddot{\tilde{\Gamma }}{}_{DD}(j,p) \end{array}\right].$$

In light of this finding, we can express $\frac{\partial^{2}{\left[Q^{\prime }(p)\right]}_{n,m}}{\partial p\partial p^{T}}$ in the matrix form as:

(A18) $$\frac{\partial^{2}{\left[Q^{\prime }(p)\right]}_{n,m}}{\partial p\partial p^{T}}=W_{1}(p)+W_{2}(p)+W_{2}^{T}(p)+W_{3}(p).$$

Then, substituting (A18) back into (A10) leads to:

(A19) ∂2[x(p)]2mL−2L+n∂p∂pT=Re{W1(p)+W2(p)+W2T(p)+W3(p)}.

Consequently, ${\ddot{X}}_{pp}(p)$ can be constructed by applying (A19) to (53).
